# Mitochondrial transfer as a driver of immune microenvironment remodeling

**DOI:** 10.3389/fimmu.2026.1743261

**Published:** 2026-04-01

**Authors:** Xiaoya Zhang, Danmei Zhang, Jin Guo, Chunxia Shi, Zuojiong Gong

**Affiliations:** Department of Infectious Diseases, Renmin Hospital of Wuhan University, Wuhan, Hubei, China

**Keywords:** cancer, immune cell, immune microenvironment, inflammation, mitochondria transfer

## Abstract

Mitochondria are central regulators of immunometabolism, and emerging evidence identifies intercellular mitochondrial transfer as a key driver of immune microenvironment remodeling. Beyond energy production, transferred mitochondria reshape immune niches by reprogramming metabolic fitness, redox balance, inflammatory tone, and immune cell interactions. Through multiple transfer routes, including tunneling nanotubes, extracellular vesicles, and gap junctions, mitochondrial exchange modulates immune activation, immunosuppression, and tolerance across diverse physiological and pathological contexts. In this review, we summarize current mechanisms of mitochondrial transfer and highlight how this process directionally remodels the immune microenvironment in inflammation, cancer, and autoimmune diseases. We further discuss therapeutic strategies aimed at modulating mitochondrial transfer to reprogram immune responses, providing new perspectives for immunomodulation and disease intervention.

## 1 Introduction

Mitochondria are essential double-membrane organelles primarily known for their role in cellular energy production through oxidative phosphorylation (OXPHOS). In contrast, glycolysis—a separate energy-generating pathway—predominantly takes place in the cytoplasm. While only OXPHOS is directly linked to mitochondrial function, both systems are critical for cellular bioenergetics. In addition to ATP synthesis, mitochondria also play key roles in calcium homeostasis, apoptosis, and regulation of intracellular redox balance (1–3). Disruptions in mitochondrial function—such as impaired ATP synthesis, excess reactive oxygen species (ROS) production, defective mitophagy, or disrupted fission–fusion dynamics—are linked to various diseases (4–7). Organs with high metabolic demands or those susceptible to hypoxia are particularly vulnerable to mitochondrial damage, underscoring the need for mitochondrial homeostasis to maintain cellular and tissue integrity.

Immune cell activation, differentiation, and effector functions are tightly dependent on mitochondrial activity and integrity (8, 9). As central regulators of immunometabolism, mitochondria orchestrate the metabolic reprogramming that underlies immune cell polarization and function (10–12). When stimulated, immune cells—including T cells, macrophages, and dendritic cells—undergo distinct metabolic transitions reflective of their activation status. For instance, activated T lymphocytes enhance mitochondrial OXPHOS to fuel clonal expansion and differentiation into effector subsets such as T helper 1 cells (Th1) and T helper 1 cells (Th2) (13). During prolonged stimulation, many immune cells gradually shift from a predominantly glycolytic metabolism toward greater dependence on mitochondrial respiration to sustain proliferation and function (14). Moreover, mitochondria act as a principal source of ROS, which serve dual roles in immunity. Controlled production of mitochondrial ROS serves as an important regulatory signal during immune responses. At physiological levels, ROS act as redox-sensitive second messengers that modulate intracellular signaling pathways involved in immune activation, including the regulation of transcription factors and cytokine expression (15). In contrast, excessive or sustained ROS accumulation disrupts redox homeostasis, leading to oxidative stress, cellular damage, and pro-inflammatory responses. Together, these observations underscore the essential role of mitochondria in fine-tuning redox balance to support effective immune signaling while preventing pathological inflammation (16).

Once confined to the cytoplasm, mitochondria are now recognized as motile organelles capable of transferring between cells. This process—termed mitochondrial transfer—occurs via various pathways, including tunneling nanotubes (TNTs), extracellular vesicles (EVs), and other cytoskeletal mechanisms (17, 18). Mitochondrial transfer has been observed in both physiological and pathological contexts across diverse cell types, including immune and tumor cells (19, 20). Exogenous mitochondria can restore membrane potential, enhance cellular respiration, and boost ATP production (21). Furthermore, mitochondrial transfer can modulate immune cell function, regulate polarization, and influence overall immune responses (22). In tumors, however, mitochondria transfer may be exploited by cancer cells to suppress immune activity and evade surveillance (23). Additionally, in immune tolerance, mitochondrial transfer may help maintain immune homeostasis (24).

Overall, mitochondrial transfer is a vital mechanism for regulating immune cell metabolism and function. By providing bioenergetic support, controlling ROS production, and modulating immune cell polarization, it shapes immune responses in both normal and disease states. As research advances, understanding mitochondrial transfer within the immune microenvironment could lead to novel therapeutic approaches for immune-mediated diseases and cancer immunotherapy. Throughout this review, we discuss mitochondrial transfer as a central mechanism driving immune microenvironment remodeling by reshaping metabolic fitness, redox balance, inflammatory tone, and immune cell interactions across physiological and pathological contexts.

## 2 Mechanisms of mitochondrial transfer

**Figure 1:**
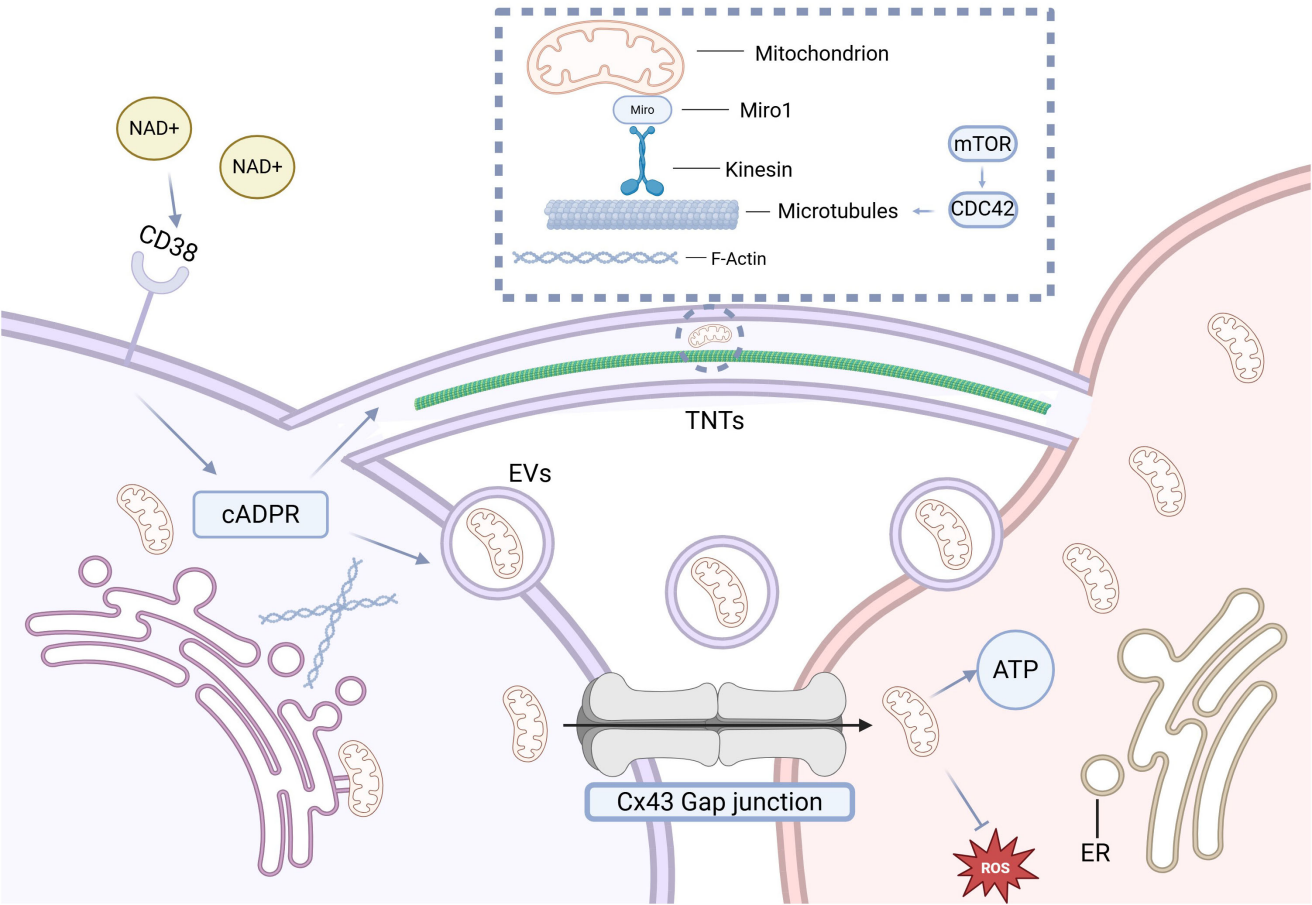
Strategies and mechanisms of MT. Mitochondria can be bidirectionally transported between cells via TNTs, GJCs, and EVs. TNT formation is driven by F-actin polymerization, and mitochondrial transport through TNTs is regulated by Miro1. EV endocytosis is mediated by the NAD^+^/CD38/cADPR pathway: under stress conditions, elevated extracellular NAD^+^ activates CD38 to generate the second messenger cADPR, which promotes EV complex activation, vesicle formation and release, as well as TNT induction. Gap junction–mediated mitochondrial transfer primarily involves Cx43, although its precise mechanism remains unclear.

Mitochondrial transfer occurs through various active mechanisms, including EVs, TNTs, gap junction channels (GJCs), and several nontraditional pathways. These mechanisms enable the transport of intact, functional mitochondria between donor and recipient cells, playing critical roles in cellular communication and function. A schematic overview of these transfer pathways is shown in **Figure 1**.

### 2.1 Extracellular vesicles

EVs represent vital mediators of cell-to-cell communication and are typically categorized according to their biogenesis and size into three main subtypes: exosomes (30–150 nm), microvesicles (100 nm–1 μm), and apoptotic bodies (>1 μm) (25). Exosomes originate from the fusion of multivesicular endosomes with the plasma membrane and primarily transport lipids, proteins, various RNA species, and occasionally mitochondrial DNA (mtDNA) (19). In contrast, microvesicles emerge directly through outward budding of the plasma membrane and possess a larger size range, allowing them to encapsulate whole, functionally active mitochondria—thereby facilitating intercellular mitochondrial exchange (26). For instance, bone marrow–derived mesenchymal stem cells (BMSCs) have been shown to deliver viable mitochondria to alveolar epithelial cells via TNTs as well as through connexin 43 (Cx43)–dependent microvesicular pathways (27). Similarly, astrocytes are capable of secreting EVs enriched with structurally intact mitochondria, which can subsequently be internalized by adjacent glial cells such as microglia (28). Notably, mitochondria-containing EVs maintain distinctive structural characteristics—including well-preserved cristae, ribosomes, and outer membrane proteins like translocase of the outer mitochondrial membrane 20 (TOMM20)—features that differentiate them from smaller mitochondrial-derived vesicles (MDVs), which generally carry only fragmented mitochondrial constituents (29, 30).

### 2.2 Tunneling nanotubes

TNTs are F-actin-based membranous protrusions that facilitate direct cytoplasmic connections between cells, enabling the transfer of various cellular materials, including mitochondria (17). These nanotubes can transport mitochondria in both unidirectional and bidirectional manners (21). TNT formation can be inhibited by actin polymerization disruptors, such as cytochalasin B, without affecting endocytosis or phagocytosis (31, 32). Interestingly, Cx43—a gap junction protein—has also been implicated in TNT biogenesis, suggesting potential crosstalk between TNTs and GJCs (33). Mitochondrial transfer along TNTs is regulated by Miro1, a key adaptor protein that coordinates mitochondrial trafficking along the actin and microtubule networks (34–36). Additionally, proteins like M-Sec and CD38 play significant roles in TNT formation and mitochondrial transfer (37–39). The overexpression of Miro1 in MSCs has been shown to enhance TNT formation and promote mitochondrial transfer to astrocytes *in vitro*, highlighting Miro1 as a potential target for enhancing intercellular communication (40). However, determining whether the phenotypic effects observed from TNT-mediated mitochondrial transfer are due solely to mitochondria or other bioactive molecules remains a challenge (41).

### 2.3 Gap junction channels

GJCs, primarily composed of connexins such as Cx43, facilitate direct exchange of ions, metabolites, and small signaling molecules between adjacent cells (42). Recent evidence suggests that GJCs also mediate mitochondrial transfer under both physiological and pathological conditions (42, 43). Notably, Cx43 is found not only on the plasma membrane but also on the outer mitochondrial membrane, where it regulates mitochondrial membrane potential, calcium flux, and oxidative stress responses (44, 45). In myocardial infarction models, BMSCs have been shown to transfer mitochondria to ischemic cardiomyocytes through a Cx43-dependent GJC mechanism, restoring ATP production and improving cell survival (46, 47). Although evidence points to Cx43 as a key player in mitochondrial exchange, the exact mechanisms of its involvement—whether independently or in coordination with TNTs—remain unclear. Nonetheless, Cx43’s dual role in intercellular communication and mitochondrial exchange positions it as a promising target for therapeutic strategies aimed at treating mitochondrial dysfunction.

### 2.4 Nontraditional mitochondrial transplantation

In addition to classical pathways such as EVs, TNTs, and GJCs, mitochondria can also be transferred through nontraditional mechanisms like cell fusion, synaptosomal exchange, and dendritic network connectivity (48–50). Cell fusion, especially between stem and somatic cells, plays an important role in tissue regeneration and somatic reprogramming (51). For instance, mitochondrial transfer has been observed when human BMSCs or adipose-derived stem cells fuse with murine cardiomyocytes (48). Experimental induction of cell fusion through Sendai virus-based systems has been shown to enhance mitochondrial transfer efficiency (52). Furthermore, unconventional routes for mitochondrial exchange, such as dendritic networks in osteocytes and synaptosomal transfer in neurons, have also been identified (49, 50). *In vivo* studies demonstrate that adipocytes can transfer mitochondria to macrophages within white adipose tissue, indicating the involvement of these pathways in tissue homeostasis and immune regulation (53).

## 3 Donors and acceptors in the immune microenvironment

**Figure 2:**
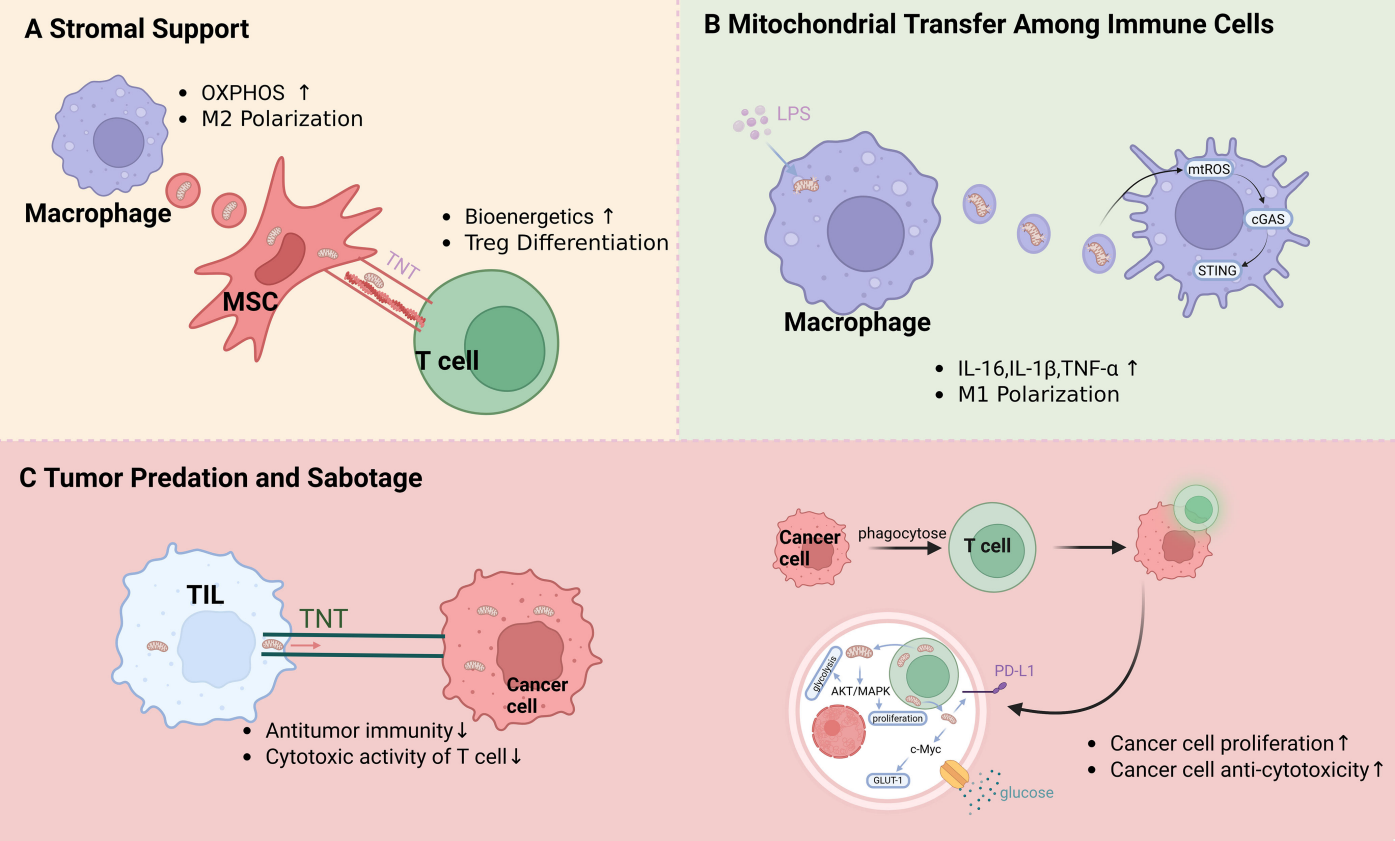
The intercellular mitochondrial transfer network in the immune microenvironment. Intercellular mitochondrial exchange establishes a highly dynamic metabolic network involving diverse donors and acceptors. **(A)** Stromal Support: MSCs donate functional mitochondria to immune cells via TNTs and EVs. This enhances OXPHOS, driving M2 macrophage polarization and Treg differentiation. **(B)** Inter-Immune Mitochondrial Transfer: During sepsis, microvesicles released by LPS-damaged macrophages are internalized by recipient macrophages. This activates the mtROS/cGAS/STING pathway, triggering an inflammatory cytokine storm. **(C)** Tumor Predation and Sabotage: Cancer cells hijack healthy mitochondria from tumor-infiltrating lymphocytes (TILs) via TNTs or cell-in-cell (CIC) engulfment. This acquisition activates AKT/MAPK signaling and upregulates c-Myc–mediated glycolysis (GLUT-1), collectively fueling tumor proliferation and elevating PD-L1 expression to facilitate immune evasion.

Intercellular mitochondrial transfer establishes a highly dynamic and intricate metabolic network within the immune microenvironment. In this ecosystem, cells act as either mitochondrial donors or acceptors, remodeling local immune responses through the strategic redistribution of bioenergetic resources. This supply-and-demand relationship encompasses unidirectional support from stromal cells, aggressive metabolic parasitism by neoplastic cells, and cooperative metabolic crosstalk among distinct immune populations to maintain homeostasis. The highly dynamic network of intercellular mitochondrial transfer within the immune microenvironment is illustrated in **Figure 2**.

### 3.1 Mitochondrial transfer from mesenchymal stem cells to immune cells

Stromal cells, particularly mesenchymal stem cells (MSCs), function as essential mitochondrial donors within the immune niche. MSCs can deliver functional mitochondria to target immune cells via EVs or TNTs, thereby augmenting the recipient’s bioenergetic capacity and modulating its functional phenotype (17, 18). For example, in models of acute lung injury, BMSCs transfer mitochondria to alveolar macrophages through Cx43-dependent gap junctions. This transfer not only restores the cellular bioenergetics of the macrophages but also significantly mitigates inflammation-induced tissue damage (27).

Stromal-to-immune mitochondrial transfer is a critical mechanism for inducing immunological tolerance. When MSCs donate mitochondria to CD3^+^ T cells or Th17 cells, they facilitate epigenetic remodeling that drives differentiation into regulatory T cells (Tregs) (54, 55). This lineage shift is stabilized by sustained FOXP3 expression, effectively restricting excessive inflammatory cascades (56).

### 3.2 Mitochondrial transfer among immune cells

Beyond stromal support and tumor-driven predation, immune cells engage in cooperative mitochondrial exchange to meet acute bioenergetic demands and rapidly coordinate immune responses. For instance, platelets serve as highly mobile donors, delivering functionally intact mitochondria to neutrophils and monocytes via EVs. This metabolic subsidy significantly elevates the oxygen consumption, ATP generation, and migratory capacity of the recipient innate immune cells (57).

The intricate communication between dendritic cells (DCs) and T cells during antigen presentation is also heavily reliant on mitochondrial dynamics (58–60). Activated T cells can prime and sensitize DCs by releasing EVs that contain both genomic and mitochondrial DNA. This antigen-driven intercellular contact triggers the cGAS-STING signaling axis and IRF3-dependent antiviral responses in the recipient DCs (61). Additionally, active mitochondrial exchange occurs among macrophages; during lipopolysaccharide (LPS)-induced inflammation, mitochondria-laden microvesicles secreted by activated macrophages can reprogram adjacent resting macrophages into a pro-inflammatory M1-like phenotype, rapidly amplifying the local immune response (62). This dynamic intercellular mitochondrial trafficking provides an indispensable metabolic foundation for tuning immune synapses and maintaining local immune efficacy.

### 3.3 Competition and predation between tumor cells and immune cells

Within the nutrient-deprived tumor microenvironment (TME), cancer cells exhibit a pronounced “metabolic parasitism,” maliciously hijacking the mitochondrial transfer network to ensure their own survival while mediating immune evasion (63). Current evidence demonstrates that neoplastic cells utilize TNTs to “steal” functional mitochondria from tumor-infiltrating T cells. This direct structural plundering results in severe metabolic suppression of the T cells, drastically impairing their cytotoxic activity and dampening antitumor immunity (64).

This predatory behavior can also manifest through extreme physical interactions, such as “cell-in-cell” (CIC) structures (65). In lung cancer, CIC formation allows malignant cells to engulf infiltrating lymphocytes entirely and hijack their mitochondrial networks. This acquisition fuels glucose metabolic reprogramming in the tumor, triggers MAPK signaling, and upregulates PD-L1 expression, thereby simultaneously accelerating tumor proliferation and disarming inflammatory immune surveillance (66).

## 4 Impact of mitochondrial transfer on immune cell reprogramming

**Figure 3:**
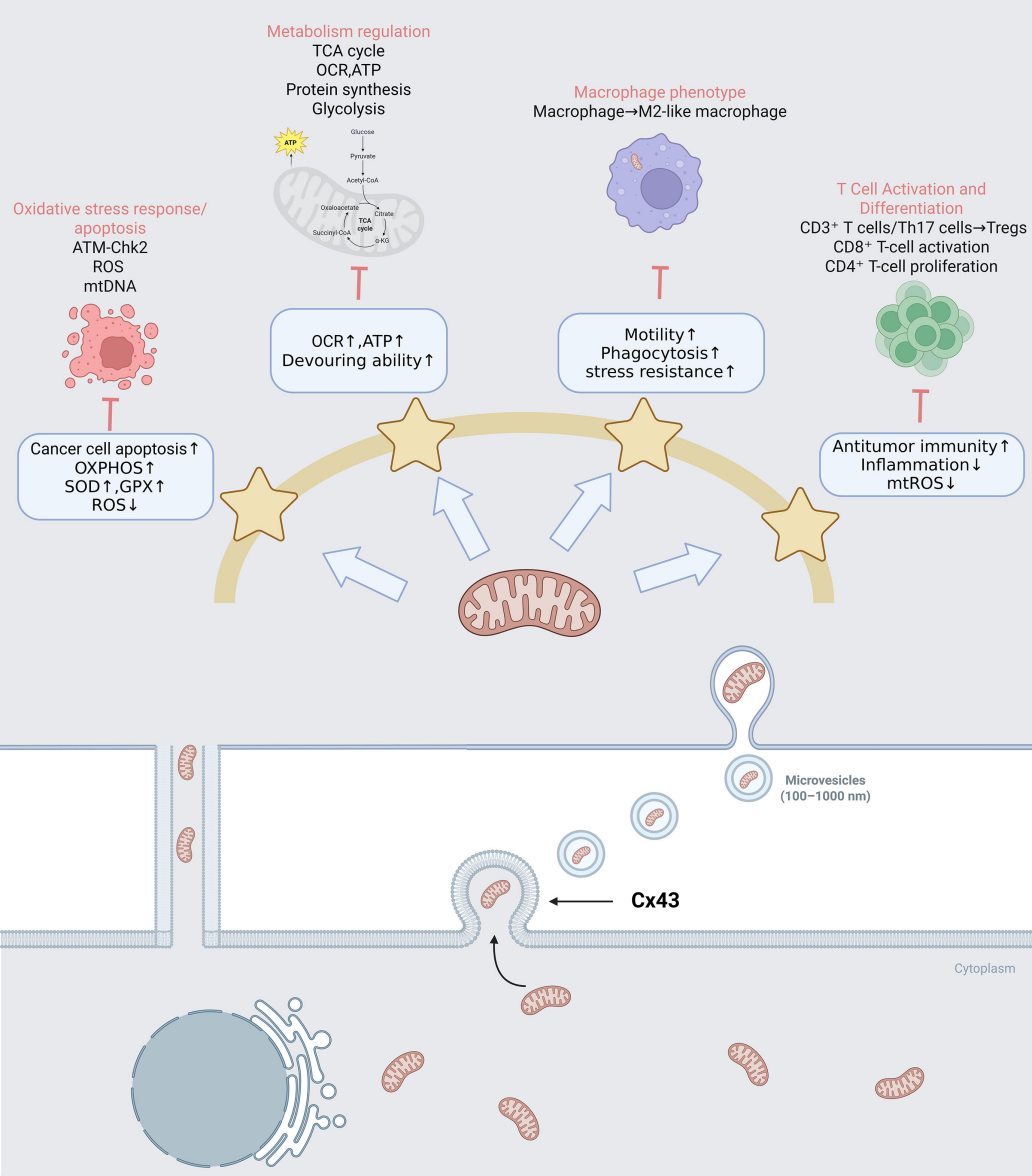
Regulatory role of mitochondrial transfer in immune cell reprogramming. Microvesicles retain essential ultrastructural features and are capable of encapsulating intact mitochondria. The transfer of healthy mitochondria from donor to recipient cells via microvesicles and TNTs effectively regulates metabolic rewiring, immune cell phenotypic switching, oxidative stress, and apoptosis.

The intercellular transfer of mitochondria functions as a profound biological modifier, fundamentally reprogramming the metabolic and functional landscape of recipient immune cells. Rather than merely supplying a transient energy burst, acquired mitochondria integrate into the host’s cellular machinery to dictate metabolic rewiring, drive phenotypic switching, and enhance cellular survival under severe stress. **Figure 3** illustrates the regulatory role of mitochondrial transfer in immune cell reprogramming.

### 4.1 Metabolic rewiring

The functional plasticity of immune cells is tightly governed by their ability to dynamically transition between glycolytic and oxidative metabolic states (1–3). The acquisition of exogenous mitochondria provides an immediate structural and biochemical foundation for this metabolic rewiring.

Specifically, the integration of functional mitochondria shifts the bioenergetic reliance of recipient cells away from glycolysis—a pathway typically associated with acute, pro-inflammatory activation—toward more efficient OXPHOS. For example, the transfer of mitochondria from MSCs to hyperactivated CD4^+^ T cells effectively curtails their glycolysis-driven activation, redirecting their metabolic profile to restore immunometabolic homeostasis (67). Within the CD8^+^ T cell compartment, TNT-mediated mitochondrial donation from BMSCs significantly amplifies the recipient’s respiratory capacity, mitochondrial mass, and overall metabolic flexibility (68). Similarly, in acute respiratory distress syndrome (ARDS), MSC-mediated mitochondrial delivery to macrophages enhances ATP turnover and phagocytic performance both *in vitro* and *in vivo* (69). This process also activates PGC-1α, a master regulator of mitochondrial biogenesis, promoting M2 macrophage polarization, suppressing inflammation, and improving energy metabolism (70).

### 4.2 Phenotypic switching

By altering the underlying metabolic circuitry, mitochondrial transfer acts as a master switch that drives definitive phenotypic transformations in both innate and adaptive immune cells.

#### 4.2.1 Macrophage phenotype

Macrophage polarization is a highly dynamic and reversible process that can be shaped by diverse microenvironmental cues, biological contexts, and disease states. Traditionally, macrophage activation has been broadly categorized into two major functional phenotypes, termed M1 and M2 macrophages (71). M1 macrophages represent an early line of defense against intracellular pathogens and promote Th1 immune responses. Upon activation, they secrete a wide array of pro-inflammatory mediators, including TNF-α, monocyte chemoattractant protein-1 (MCP-1), interleukins such as IL-1, IL-6, and IL-12, type I interferon (IFN-1), inducible nitric oxide synthase (iNOS), as well as multiple C–X–C motif chemokine ligands (CXCLs), including CXCL1–3, CXCL5, and CXCL8–10 (72). In contrast, M2 macrophages are generally associated with anti-inflammatory functions and tissue remodeling. This group encompasses several subtypes—M2a, M2b, M2c, and M2d—each characterized by distinct surface markers, cytokine profiles, and functional properties. While M2a macrophages primarily support tissue repair and regeneration, M2b, M2c, and M2d subsets play important roles in immune regulation, phagocytosis, and tumor-associated processes, respectively (73, 74). Notably, M2d macrophages constitute a major component of the tumor microenvironment and are commonly referred to as tumor-associated macrophages (TAMs) (75, 76), where they have been implicated in promoting tumor progression, invasion, and immune suppression (77). Importantly, emerging evidence indicates that mitochondrial transfer can reprogram macrophage functional states by reshaping cellular metabolism, mitochondrial adaptability, and inflammatory signaling outputs (53). Through these mechanisms, mitochondrial transfer profoundly influences macrophage behavior and contributes to the dynamic regulation of inflammatory tone within the immune microenvironment.

In myocardial infarction, mitochondrial delivery drives macrophages toward an M2-like reparative phenotype with improved motility, phagocytosis, and stress resistance (78). In bacterial challenge models, MSCs transfer mitochondria to macrophages via TNTs, thereby enhancing antimicrobial capacity (69). These vesicles also carry regulatory microRNAs (miR-451, miR-1202, miR-630, miR-638) that downregulate TLRTIL expression, suppressing mtDNA-induced inflammation and fostering immune tolerance (79). Nonetheless, mitochondrial transfer is not universally beneficial. In melanoma, exogenous mitochondrial transplantation enhances tumor progression by promoting M2-like macrophage accumulation through Nrf2/HO-1 signaling (80).

Recent evidence indicates that M2d macrophage often display fragmented mitochondrial networks and an increased propensity to transfer mitochondria to adjacent cancer cells. These transferred mitochondria accumulate ROS in recipient tumor cells and activate ERK signaling in a ROS-dependent manner, thereby promoting tumor progression (81). Notably, current studies investigating mitochondrial transfer in macrophages have largely focused on its role in shaping classical M1- and M2-like polarization states, whereas its effects on other macrophage phenotypes and functional subsets remain poorly characterized. As macrophage activation is increasingly recognized as a dynamic and heterogeneous continuum, future studies should explore how mitochondrial transfer modulates distinct macrophage subsets under diverse physiological and pathological conditions.

#### 4.2.2 T cell activation and differentiation

CD8^+^ cytotoxic T lymphocytes (CTLs) are central to antiviral and antitumor immunity. Upon antigen encounter, naïve CD8^+^ T cells proliferate and differentiate into effector CTLs under the influence of T-cell receptor (TCR) activation, co-stimulatory signaling, and cytokine cues, supported by CD4^+^ helper T-cell–derived IL-2 and IL-21 (82, 83). Mitochondrial dysfunction contributes to T-cell exhaustion, whereas mitochondrial transfer restores bioenergetic activity and reinvigorates exhausted populations (35, 84). Notably, MSC-derived mitochondrial transfer can exert immunosuppressive effects by suppressing the transcription factors T-bet and Eomes, thereby attenuating IFN-γ secretion (85). Furthermore, this organelle exchange facilitates the induction of a tolerogenic phenotype by upregulating Treg-specific signatures (e.g., FOXP3, IL2RA, CTLA4), promoting the expansion of suppressive CD25^+^FoxP3^+^ populations (86).

Age-related decline in CD4^+^ T-cell mitochondrial function contributes to immunosenescence. Introducing mitochondria from embryonic fibroblasts into aged CD4^+^ T cells enhances proliferation, reduces mitochondrial ROS, increases antioxidant expression, and improves oxidative metabolism (87). Similar rejuvenating effects have been observed in aged human CD4^+^ T cells (88). Furthermore, disruption of MSC-derived EV transfer impairs their ability to metabolically reprogram activated CD4^+^ T cells, underscoring the functional importance of this exchange (89). MSC-mediated mitochondrial transfer also suppresses Th1 proliferation and IFN-γ release by downregulating T-bet (90). Moreover, mitochondrial ROS support plasmacytoid DC cross-presentation, enhancing CD8^+^ T-cell activation and strengthening antitumor immunity (91).

### 4.3 Mitigating oxidative injury and apoptosis

Beyond modulating immune effector functions, mitochondrial transfer serves as a robust cytoprotective mechanism that ensures immune cell survival in hostile microenvironments. A primary survival benefit is the rapid buffering of oxidative stress. The integration of healthy mitochondria reestablishes redox equilibrium, significantly diminishing the pathological accumulation of intracellular ROS (92). This restoration is accompanied by a marked upregulation in the activity of crucial antioxidant enzymes, including superoxide dismutase (SOD) and glutathione peroxidase (GPX), which collaboratively neutralize oxidative tissue damage (93). This ROS-scavenging capability is particularly vital for preventing cellular senescence and functional decline in aged CD4^+^ T cells, as well as for limiting excessive activation and inducing protective mitophagy in B lymphocytes (87, 94).

Ultimately, this metabolic rescue directly dictates cell survival by regulating apoptotic cascades. By repairing the mitochondrial network, MSC-mediated transfer effectively suppresses terminal apoptotic pathways and preserves the viability of severely damaged recipient cells, such as glutamate-injured neurons in neurodegenerative models (95). Interestingly, in highly specific oncology contexts, the introduction of healthy mitochondria can reverse the apoptotic evasion typical of cancer cells, resensitizing them to ROS-dependent apoptosis via the PI3K/AKT and targeted caspase cascades (96).

## 5 Mitochondrial transfer in remodeling the pathological immune microenvironment

Immune cells depend not only on their own mitochondria for sustaining energy metabolism and functional activity but also engage in intercellular mitochondrial transfer. This exchange allows immune cells to communicate with one another, modulating the immune microenvironment by coordinating inflammatory signaling, cytokine production, and redox balance.

### 5.1 Dual roles of mitochondrial transfer in remodeling the inflammatory niche

**Figure 4:**
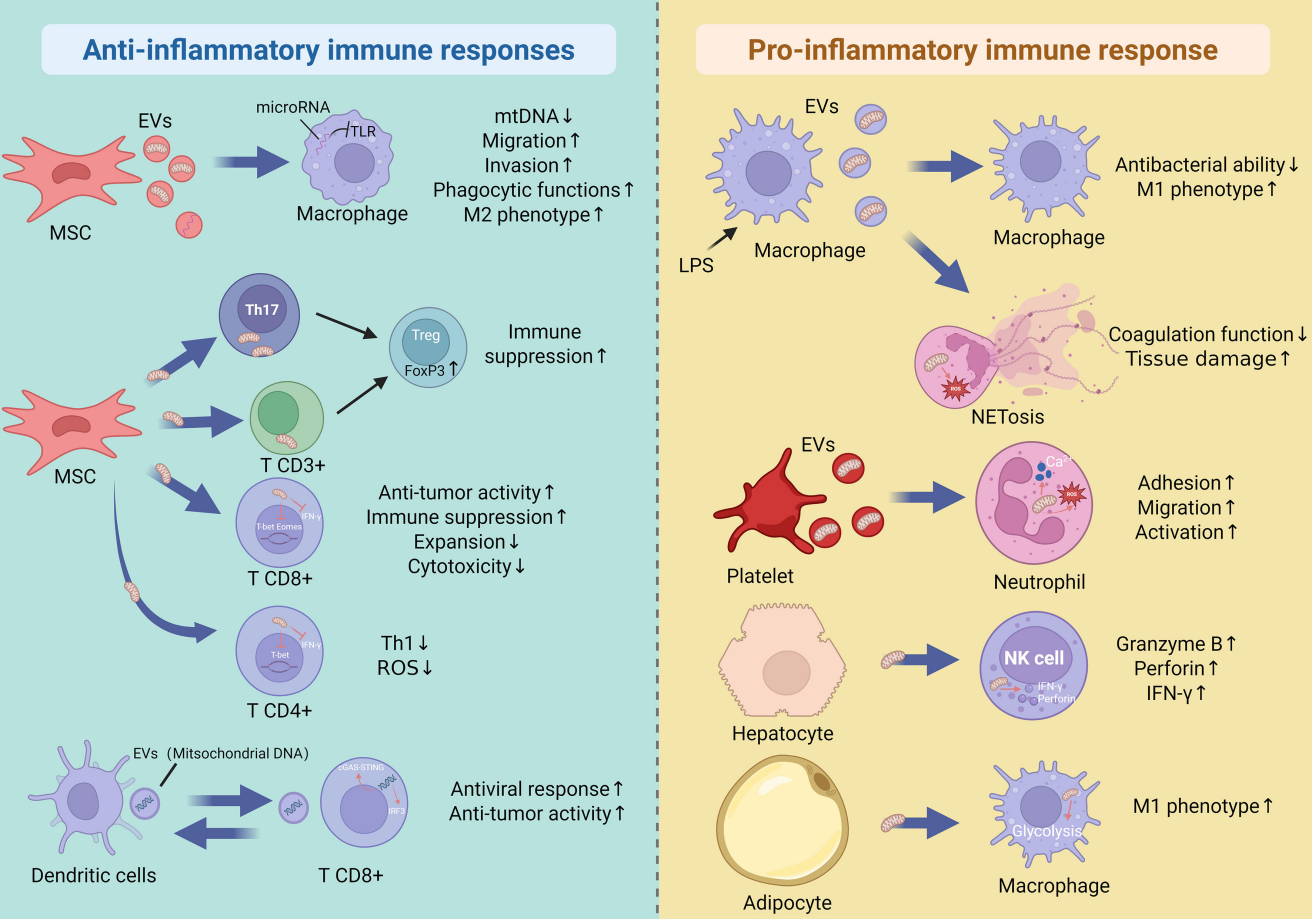
Dual roles of mitochondrial transfer in inflammation. Mitochondrial transfer from MSCs to macrophages drives M2 polarization and anti-inflammatory responses. Mitochondria transferred from MSCs to Th17 or CD3^+^ T cells promote epigenetic reprogramming toward the Treg lineage via FOXP3 activation, while those delivered to CD4^+^ T cells suppress Th1 proliferation and IFN-γ production through T-bet inhibition. Conversely, under LPS stimulation, mitochondria transferred to macrophages or neutrophils enhance mtROS production and NET formation, contributing to inflammation. Platelet- and hepatocyte-derived mitochondria enhance neutrophil activation and NK cell cytotoxicity, respectively. Under metabolic stress, adipose tissue macrophages utilize exogenous mitochondria to sustain aerobic respiration and thermogenesis, thereby promoting a pro-inflammatory phenotype.

Mitochondrial transfer is a key mechanism regulating immune cell behavior, functioning as a context-dependent biological rheostat that dictates microenvironmental outcomes. Depending on the donor cell type, the functional integrity of the transferred mitochondria, and the local metabolic cues, this intercellular exchange can either drive robust pro-inflammatory effector functions or enforce anti-inflammatory resolution and tissue repair. **Figure 4** illustrates the bidirectional regulation of these immune responses.

#### 5.1.1 Establishment of the pro-inflammatory niche

During the initiation of an immune response, the microenvironment requires a massive bioenergetic surge to support pathogen clearance and cytotoxicity. In this context, mitochondrial transfer acts as a metabolic accelerant to establish a pro-inflammatory niche.

For innate immunity, the acquisition of mitochondria is often a prerequisite for effective pathogen clearance. Platelets have been shown to transfer mitochondria to neutrophils and monocytes via EVs, a process that markedly enhances their oxygen consumption and ATP production (57). During severe inflammatory states such as sepsis, microvesicles released from pyroptotic macrophages trigger neutrophil extracellular trap (NET) formation via a process dependent on mitochondrial GSDMD-N, thereby promoting tissue injury and coagulopathy (97). Macrophages themselves are also subject to this pro-inflammatory remodeling. In lipopolysaccharide (LPS)-induced inflammation, mitochondria-containing microvesicles derived from activated macrophages can reprogram neighboring resting macrophages into an M1-like phenotype, amplifying local tissue injury (62). Additionally, under metabolic stress, the inhibition of mitochondrial transfer by LCFAs forces adipose tissue macrophages to shift toward glycolysis, favoring a pro-inflammatory M1-like profile (53, 98). Communication between T cells and DCs may involve EV-mediated transfer of genomic and mitochondrial DNA, triggering cGAS–STING signaling and IRF3-dependent antiviral responses (61). Furthermore, natural killer (NK) cells rely heavily on mitochondrial fitness for optimal immune surveillance; mitochondrial supplementation from allogeneic sources significantly enhances their proliferation and effector functions, including the secretion of granzyme B, perforin, and IFN-γ (99, 100).

#### 5.1.2 Transition to the anti-inflammatory and reparative niche

Conversely, to prevent excessive host damage and restore homeostasis, mitochondrial transfer actively orchestrates the transition from inflammation to resolution. In this scenario, acquired mitochondria serve as a “metabolic brake” that suppresses effector activation and induces regulatory phenotypes.

MSCs are master regulators of this anti-inflammatory remodeling. MSC-derived EVs carrying functional mitochondria enhance macrophage phagocytic capacity while suppressing the release of pro-inflammatory cytokines, effectively driving macrophages toward an M2-like reparative phenotype (101). Similarly, mitochondria-containing EVs from adipose-derived stem cells promote M2-like polarization, which is crucial for debris clearance and wound healing (102). In clinical disease models, such as ARDS and myocardial infarction, this directed mitochondrial delivery drives macrophages toward an M2-like state, improving stress resistance, dampening inflammation, and facilitating tissue repair (101, 103).

This suppressive remodeling also profoundly restrains adaptive immunity. Mitochondrial transfer from MSCs serves as a potent tolerogenic signal, reprogramming Th17 cells and CD3^+^ T cells toward Treg phenotype by stabilizing FOXP3 expression (54). MSC-mediated transfer also upregulates Treg-related transcripts such as IL2RA, CTLA4, and TGF-β1, driving the expansion of immunosuppressive CD25^+^FoxP3^+^ T cells (86). Concurrently, this exchange suppresses Th1 proliferation and downregulates the transcription factors T-bet and Eomes, significantly reducing IFN-γ production and enforcing immunosuppression (85, 90). Beyond T cells, mitochondrial transfer from MSCs to B cells reduces ROS accumulation and induces mitophagy, thereby limiting B-cell activation and pro-inflammatory cytokine production (94). In aging models, introducing exogenous mitochondria into senescent CD4^+^ T cells improves oxidative metabolism, diminishes ROS, and rejuvenates their homeostatic functions, counteracting age-related immunosenescence (87, 88).

### 5.2 Tumor immune microenvironment

Mitochondrial transfer within the TME is increasingly recognized as a dual-purpose mechanism for metabolic adaptation and immune subversion. By depleting the bioenergetic reserves of TILs, tumor cells induce a state of metabolic exhaustion that facilitates evasion (104). This “organelle theft” impairs the functional maturation of NK and T cells, while the subsequent cytosolic release of acquired mtDNA can hijack the cGAS-STING pathway to promote pro-tumorigenic interferon signaling (105). This dynamic competition underscores the pivotal role of mitochondrial flux in determining the TME’s inflammatory tone. Furthermore, tumors can transfer dysfunctional mitochondria carrying mutated mtDNA into T cells. Because the TME is often enriched with mitophagy inhibitors like USP30, T cells are unable to clear these damaged organelles, triggering metabolic collapse, cellular senescence, and terminal immune exhaustion (106, 107).

Highly metastatic tumor cells can also disseminate mitochondria containing pathogenic mtDNA to less aggressive cells via EVs, promoting widespread invasiveness (103). Beyond EVs and TNTs, tumors can acquire mitochondria from stromal partners such as cancer-associated fibroblasts (CAFs) and MSCs, enhancing metabolic flexibility and resistance to both immune attack and therapy (108, 109). Similarly, adipose-derived stem cells (ASCs) deliver mitochondria to tumor cells through TNTs and gap junctions, sustaining cancer cell metabolism and survival under stress (110). In the brain, tumor-initiating cells have been shown to transfer mitochondria to astrocytes, impairing their supportive function and promoting malignancy (111). In breast cancer, mitochondrial transfer predominantly occurs through TNTs between mammary epithelial and tumor cells, reprogramming tumor metabolism and facilitating microenvironmental remodeling (92). Emerging evidence indicates that osteocytes can donate mitochondria to metastatic cancer cells, leading to activation of the cGAS-STING signaling pathway and subsequent induction of antitumor immune responses. This mitochondrial transfer enhances tumor immunogenicity and effectively restrains the establishment and progression of metastatic cancer cells within the bone microenvironment (112).

**Figure 5:**
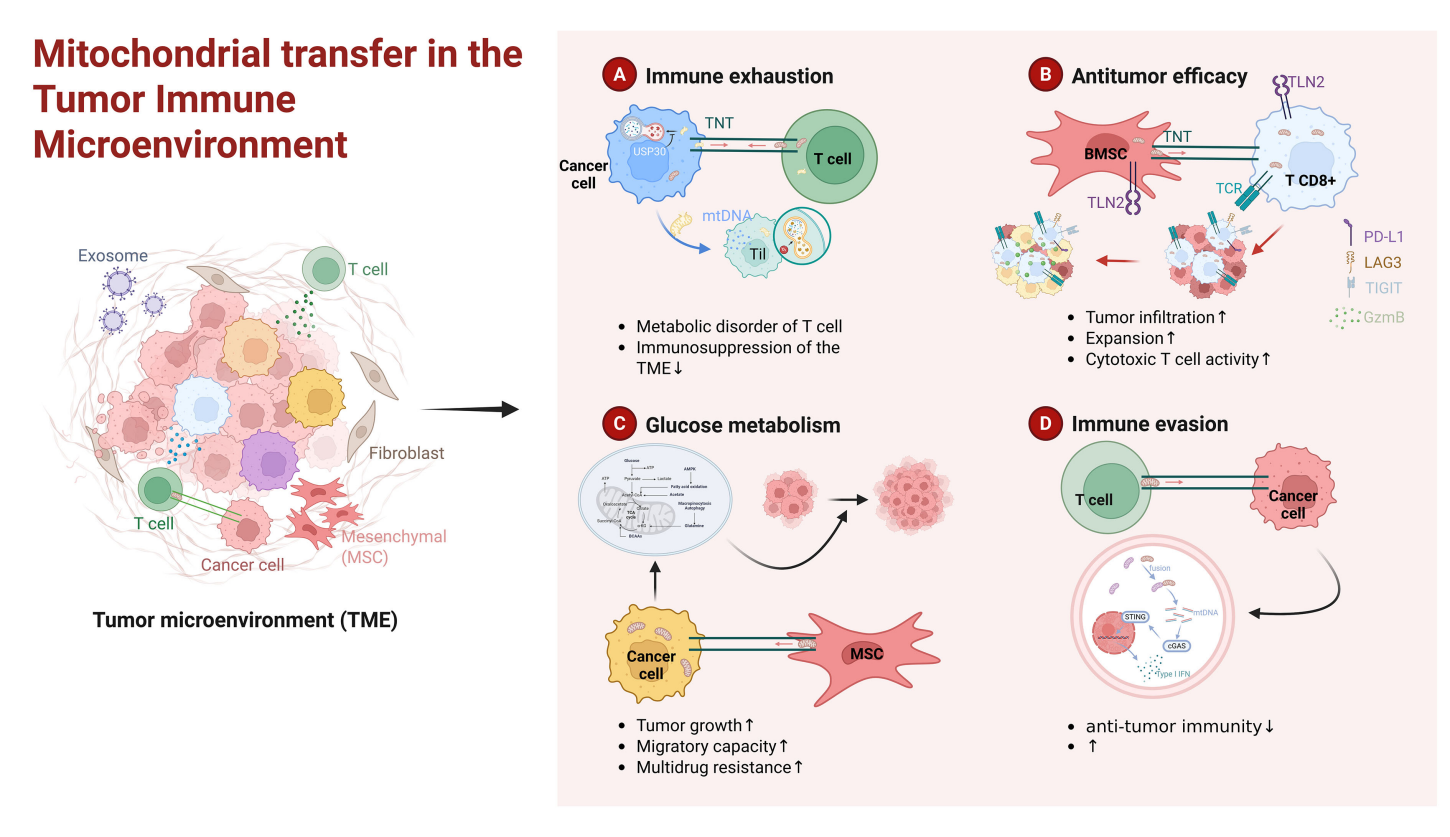
Mitochondrial transfer within the tumor microenvironment. **(A)** Cancer cells can acquire mitochondria from T cells via TNTs, thereby suppressing antitumor immunity. In addition, mitochondria from cancer cells carrying mtDNA mutations can be transferred to lymphocytes, where inhibition of mitophagy by USP30 allows the persistence of dysfunctional mitochondria, leading to T-cell metabolic imbalance and senescence. **(B)** Mitochondrial transfer from BMSCs enhances CD8^+^ T-cell proliferation and infiltration while downregulating the expression of inhibitory receptors PD-1, LAG3, and TIGIT. **(C)** Conversely, mitochondria transferred from MSCs to cancer cells via TNTs promote tumor cell survival and metabolic activity. Metabolically impaired cancer cells obtain mitochondria from MSCs to sustain their bioenergetic demands and thrive within the TME. **(D)** Cancer cells acquire mitochondria from immune cells, a loss that compromises innate and adaptive antitumor immunity. The fusion of these hijacked mitochondria with endogenous tumor mitochondria activates the cGAS-STING pathway, which subsequently drives tumor invasion through type I interferon signaling.

Recent single-cell analyses using Mitochondria-Enabled Reconstruction of Cell Interactions (MERCI) have traced mitochondrial transfer events in human tumors, revealing associated gene networks linked to cytoskeletal remodeling, oxidative metabolism, and TNF-α-driven inflammatory signaling (113). **Figure 5** depicts how mitochondrial transfer acts both as a means of metabolic adaptation and as a covert immune evasion mechanism within the TME. These insights open avenues for targeting mitochondrial dynamics and intercellular exchange in cancer immunotherapy.

### 5.3 Autoimmune diseases

Mitochondrial integrity serves as a cornerstone for maintaining immunological self-tolerance and systemic equilibrium. Beyond their canonical role in ATP production, mitochondria function as sophisticated signaling hubs that dictate immune cell fate and lineage stability. The maintenance of Treg cell fitness, in particular, is intrinsically linked to mitochondrial bioenergetics, structural plasticity, and redox homeostasis (114). Precise modulation of mitochondrial stress responses is indispensable for sustaining FOXP3 expression, while mitochondrial-derived metabolites exert epigenetic control over Treg differentiation; conversely, mitochondrial decay precipitates metabolic dysregulation and impairs the suppressive repertoire of Tregs, thereby fueling autoimmune onset (115, 116).

The role of mitochondria in the autoimmune landscape is fundamentally characterized by a functional dichotomy. Under homeostatic conditions, the mitochondrial-derived vesicle (MDV) pathway facilitates quality control by sequestering damaged components. However, under pathological stress, the aberrant release of mitochondrial constituents—acting as damage-associated molecular patterns (DAMPs)—can subvert immune tolerance. The leakage of mtDNA into the cytosol triggers the cGAS–STING or TLR9 pathways, catalyzing chronic inflammatory cascades (117). In conditions such as rheumatoid arthritis (RA) and systemic lupus erythematosus (SLE), EVs laden with oxidized mitochondrial cargo and nucleic acids drive the production of IFN-I via the activation of NLRP3 inflammasomes and TLR4 signaling, thereby exacerbating tissue destruction (118–123). Furthermore, external insults, such as heavy metal toxicity (e.g., cadmium), can aggravate these processes by disrupting dynein-mediated mitochondrial trafficking and perturbing organelle dynamics (124).

**Figure 6:**
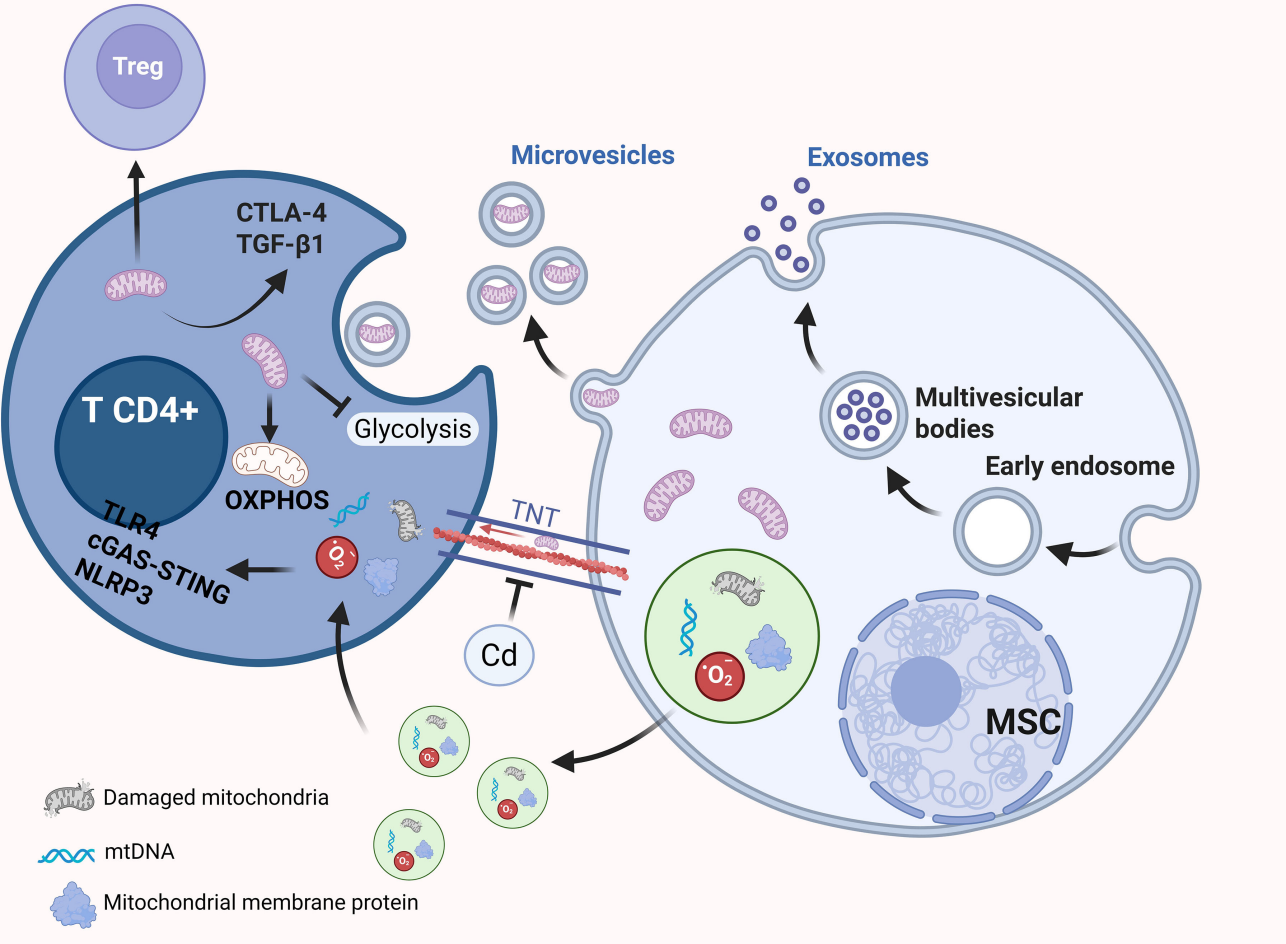
Regulation of immune tolerance. Mitochondrial transfer from MSCs to CD4^+^ T cells can induce the differentiation of FOXP3^+^ Tregs, accompanied by the upregulation of CTLA-4 and TGF-β1. In addition, MSC-derived EVs can deliver mitochondria to metabolically reprogrammed CD4^+^ T cells, where the suppression of glycolysis promotes OXPHOS. Cd disrupts the interaction between mitochondria and motor proteins such as dynein, thereby inhibiting mitochondrial transport. Cells can also package mitochondrial components—including mtDNA, mitochondrial membrane proteins, and metabolic enzymes—into EVs via the MDV pathway. Under stress or pathological conditions, damaged or oxidized mitochondrial contents released through mitoEVs can be recognized by immune cells, activating inflammatory signaling pathways such as TLR4, cGAS–STING, and NLRP3.

Despite their potential to trigger inflammation, the horizontal transfer of intact, functional mitochondria offers a transformative therapeutic paradigm for resetting the immune microenvironment. Evidence from autoimmune hepatitis (AIH) models demonstrates that MSC-derived EVs can deliver healthy mitochondria to reprogrammed CD4^+^ T cells, effectively reversing glycolysis-heavy activation and mitigating hepatic injury (89). Similarly, in multiple sclerosis (MS) and EAE models, MSCs leverage mitochondrial transfer and autophagy to restore the Th17/Treg balance, subsequently suppressing neuroinflammation and promoting remyelination (125). By fusing with the endogenous network of recipient cells, these exogenous mitochondria resuscitate oxidative metabolism and alleviate cellular stress (126). Collectively, these insights underscore that the strategic delivery of functional mitochondria represents a potent bioenergetic intervention capable of recalibrating immune responses and promoting the resolution of autoimmune diseases. **Figure 6** illustrates the mechanisms by which mitochondrial transfer contributes to the pathogenesis and regulation of autoimmune diseases.

## 6 Therapeutic strategies targeting mitochondrial transfer

Mitochondrial transfer faces several clinical challenges, particularly regarding mitochondrial heterogeneity and immunocompatibility. When exogenous mitochondria enter recipient cells, they coexist with endogenous mitochondria, forming a heteroplasmic population. This can cause mitochondrial-nuclear genome incompatibility, potentially impairing energy metabolism and long-term stability. Another challenge is optimizing transfer efficiency. Although some cell populations, such as highly purified MSC clones (RECs), exhibit better transfer capacity, free mitochondria entering target cells remain inefficient. **Table 1** summarizes current strategies to modulate or utilize mitochondrial transfer for therapy.

**Table 1:** Therapeutic strategies targeting mitochondrial transfer.

Specific approach	Mechanism of action	Target population	Key efficacy data	References
Delivery systems				
ROCK inhibitor Y-27632	Increased formation of TNT	ARPE19 cells	Increased mitochondrial movement, enhanced mitochondrial transfer	(127)
STING-activating nanofactories	Regulates mitochondrial dysfunction	Exhausted T cells	Restored immune activation	(128)
The sequential treatment of pioglitazone and iron oxide nanoparticles	High-powered mesenchymal stem cells with promoted mitochondrial biogenesis and facilitated mitochondrial transfer	lung epithelial cells	Promoted mitochondrial biogenesis, facilitated mitochondrial transfer	(129)
molybdenum disulfide (MoS2)	Enhances mitochondrial function	hMSCs	Enhanced transfer efficiency	(130)
Mitophagy agonist nanofactories	Hydrogel co-delivery with CAR-T cells	CAR-T cells in solid tumors	Enhanced memory formation	(131)
Recombinant human collagen III protein	Human umbilical cord mesenchymal stem cells (hUC-MSCs) derived Evs	L929 cells	Skin wound healing	(132)
Metabolic modulators				
Nicotinamide riboside (NR)	NAD+ precursor mitochondrial restoration	Exhausted T cells	Enhanced mitochondrial fitness	(133)
P4HA1 targeting	Enhances mitochondrial function	CD8^+^ T cells	Enhanced antitumor immunity	(134)
Bezafibrate	PPAR agonist mitochondrial targeting	CD8^+^ T cells	Improved tumor infiltration	(135)
Inhibition strategies				
TNT formation inhibitors	Cofilin pathway targeting, Actin polymerization inhibitor	Cancer-immune interactions	Reduced pathological transfer	(35, 136, 137)
EV release inhibitors	Targeting P2X7 receptor	Detrimental transfer contexts	Blockade of pathological transfer	(138)
Optical manipulation	Near-infrared control of TNTs	Precision cell targeting	Selective transfer inhibition	(139)

### 6.1 Delivery systems

Intercellular mitochondrial transfer is increasingly recognized as a key biological mechanism that supports tissue repair, regeneration, and disease mitigation (18, 19, 38). Pharmacologic modulation has been shown to enhance this process. For instance, the ROCK inhibitor Y-27632 promotes TNTs formation and mitochondrial exchange in retinal pigment epithelial cells (127). In the TME, T-cell exhaustion limits the efficacy of manganese-based STING-activated cancer immunotherapy. The co-delivery of manganese (Mn) and spermidine (SPD) activates the STING pathway in dendritic cells, alleviates hypoxia in the TME, and effectively reverses CD8^+^ T-cell exhaustion, thereby improving the outcomes of Mn-based immunotherapy (128).

MSCs are currently the leading candidates for mitochondrial transplantation, yet the natural transfer efficiency between cells remains low. A combined engineering approach—using Pg to induce mitochondrial biogenesis and iron oxide nanoparticles (IONPs) to enhance Cx43 expression—has been shown to synergistically improve mitochondrial transfer (129). Similarly, nanomaterials such as molybdenum disulfide (MoS_2_) nanoflowers with atomic-level vacancies stimulate mitochondrial biogenesis, doubling mitochondrial mass and increasing transfer frequency, thereby boosting ATP synthesis and respiration in recipient cells (130).

Recent advances in biomaterials have enabled the incorporation of EVs into scaffolds to improve delivery efficiency. Anchoring EVs to scaffold surfaces or embedding them during fabrication enhances their stability, bioactivity, and controlled release (140). Hydrogels have proven particularly effective for EV-based therapies. For instance, injectable hydrogels co-delivering CAR-T cells and the mitophagy activator BC1618 significantly improved outcomes in triple-negative breast cancer (TNBC) (131). Recombinant human collagen type III (rhCol III) hydrogels loaded with EVs have also promoted macrophage polarization toward the M2 phenotype, stimulated fibroblast migration, and enhanced angiogenesis in endothelial cells, suggesting broad potential in wound repair (132). Moreover, MSCs encapsulated within crosslinked gelatin methacryloyl (GelMA) hydrogels reduce oxidative stress and promote mitochondrial fusion through active mitochondrial transfer, alleviating mitochondrial damage and inflammation (141).

### 6.2 Metabolic modulators

Mitochondrial transfer—whether mediated through TNTs, EVs, or cell fusion—is often triggered by metabolic stress such as hypoxia, excessive ROS, ATP depletion, or calcium imbalance (142). Targeting mitochondrial metabolism and preserving mitochondrial fitness in immune cells, particularly CD8^+^ T cells, is essential to improve the efficacy of immunotherapy. Nicotinamide riboside (NR) supplementation has been shown to prevent T-cell exhaustion, maintain mitochondrial integrity, and enhance therapeutic outcomes (133). Accumulation of prolyl-4-hydroxylase subunit alpha-1 (P4HA1) disrupts the tricarboxylic acid (TCA) cycle through aberrant α-ketoglutarate and succinate metabolism, promoting mitochondrial dysfunction and T-cell exhaustion. Inhibiting P4HA1 restores the expansion of TCF1^+^ CD8^+^ T-cell progenitors across tumors and lymphoid tissues, producing durable systemic antitumor immunity (134). Likewise, the PPARα agonist fenofibrate boosts mitochondrial activity, increases CD8^+^ T-cell infiltration, and reduces myeloid-derived suppressor cells (MDSCs), markedly suppressing lung tumor progression and metastasis (135).

### 6.3 Inhibition strategies

Although mitochondrial transplantation exhibits substantial therapeutic promise in experimental models of inflammation and cancer, its clinical application remains constrained by several factors. Maintaining mitochondrial integrity *in vivo*—especially under high-calcium or inflammatory conditions—is crucial to prevent structural and functional deterioration (143). Furthermore, the optimal transplantation strategy may vary among tumor types, emphasizing the need for personalized, context-specific approaches (144).

Mitochondrial transfer can be blocked at multiple levels. Gap junction–mediated transfer can be inhibited using connexin-targeting agents such as 18-α-glycyrrhetinic acid, whereas vesicular transfer can be reduced by the dynamin inhibitor dynasore (137). Since cofilin regulates actin remodeling required for TNTs formation, inhibiting cofilin suppresses oxidative stress–induced TNTs generation (136). Similarly, microtubule-targeting agents such as taxanes and vinca alkaloids disrupt mitochondrial transport by impairing cytoskeletal dynamics (145). Actin polymerization inhibitors, including cytochalasin B (CytoB), cytochalasin D (CytoD), and metformin, further reduce TNTs formation and mitochondrial trafficking (35, 137).

Extracellular ATP (eATP) can activate P2X7 receptors (P2X7R), triggering the release of mitochondria-enriched microvesicles. These vesicles can integrate into recipient mitochondrial networks in a P2X7R-dependent manner, concurrently transferring NLRP3 and P2X7R, which promote ATP synthesis and pro-inflammatory signaling (138). Recently, optical manipulation techniques have emerged to modulate mitochondrial dynamics with high precision. By propagating near-infrared light through TNTs, researchers have successfully directed the movement of individual mitochondria between cells. This TNTs-based photonic delivery system can interrupt tumor-mediated mitochondrial hijacking from immune cells, thereby restoring immune cell function and suppressing tumor growth (139).

## 7 Conclusions and outlook

Mitochondrial transfer has gained recognition as a novel form of intercellular communication that profoundly influences immune regulation and disease progression. Beyond their canonical role as the cell’s “powerhouse,” mitochondria orchestrate immunometabolic remodeling and determine cellular fate. Functional mitochondria can be exchanged among cells via TNTs, EVs, and gap junctions, exerting effects on immune homeostasis, inflammation, tumor immunity, and tissue regeneration.

Under pathological conditions, mitochondrial damage, mtDNA leakage, and excessive ROS can activate innate immune sensors—such as the cGAS–STING, NLRP3, and TLR9 pathways—driving chronic inflammation and autoimmunity. In contrast, MSCs can alleviate oxidative stress, promote the differentiation of Tregs, and restore immune tolerance through mitochondrial transfer, offering novel therapeutic insights for autoimmune diseases such as RA, SLE, AIH, and MS.

In cancer, mitochondrial transfer exhibits dualistic behavior. Tumor cells may hijack mitochondria from immune cells or transfer defective mitochondria to lymphocytes, inducing metabolic exhaustion and facilitating immune evasion. Conversely, transfer of healthy mitochondria from MSCs or BMSCs can restore metabolic balance and enhance antitumor activity in CD8^+^ T cells and dendritic cells. This bidirectional dynamic underscore the central role of mitochondrial exchange in maintaining immunometabolic equilibrium within the TME.

Despite these promising findings, many mechanistic aspects remain to be clarified. Key challenges include controlling the directionality of mitochondrial exchange, ensuring mitochondrial stability *in vivo*, and minimizing immunogenic or pro-inflammatory risks. Future research should focus on unraveling the molecular regulators of transfer efficiency, establishing reliable tracking methods beyond fluorescent labeling, and developing standardized quality-control protocols to ensure reproducibility across studies.

Integrating mitochondrial transfer modulation with current immunotherapies offers a highly promising avenue for overcoming therapeutic resistance and improving patient outcomes. A deeper understanding of the interplay among mitochondrial transfer, metabolic rewiring, and immune signaling will not only elucidate disease mechanisms but also pave the way for innovative therapeutic strategies.
